# POLICY SERIES: REEFER MADNESS, SADNESS, OR GLADNESS? THE CANNABIS AND OLDER PERSONS STUDY

**DOI:** 10.1093/geroni/igz038.735

**Published:** 2019-11-08

**Authors:** Brian P Kaskie, Julie Bobitt

**Affiliations:** 1 University of Iowa, College of Public Health, Iowa City, Iowa, United States; 2 University of Illinois, Champaign-Urbana, Illinois, United States

## Abstract

In 2016, we began our examination of the intersection between cannabis and older persons by convening focus groups with 163 older adults from senior centers and dispensaries in nine states with varying levels of cannabis legalization. Since then, we have secured competitive research grants and contracts to examine cannabis use among older persons in California, Colorado, Illinois and Iowa. Our work is guided by the primary hypothesis that cannabis use among older persons is shaped by an individual’s calculations concerning risk (e.g., developing a cannabis use disorder, lawbreaking) and reward (e.g., relaxation, symptom relief), and individuals living in a state with a legal cannabis program may perceive less risk and also may be receiving more information about the benefits of cannabis. We also hypothesize that older adults’ access to and use of cannabis is shaped by where they live, particularly defined by local cannabis program implementation efforts and relevant contextual conditions. In this symposium, we will examine our latest work concerning (a) life-span attitudes toward cannabis, (b) clinical perspectives on counseling and certifying older persons for medical cannabis, (c) provider perspectives on state cannabis policy and program implementation, (d) cannabis use among a sample of dementia caregivers and (e) outcomes experienced by older persons who use cannabis for medical or recreational purposes. Our discussion focuses on implication for policy development and program implementation.

